# A longitudinal study of the durability of long-lasting insecticidal nets in Zambia

**DOI:** 10.1186/s12936-016-1154-4

**Published:** 2016-02-19

**Authors:** Kathrine R. Tan, Jane Coleman, Barbara Smith, Busiku Hamainza, Cecilia Katebe-Sakala, Casey Kean, Ashley Kowal, Jodi Vanden Eng, Tiffany K. Parris, Carla T. Mapp, Stephen C. Smith, Robert Wirtz, Mulakwa Kamuliwo, Allen S. Craig

**Affiliations:** Center for Global Health, Malaria Branch, Centers for Disease Control and Prevention, 1600 Clifton Rd. MS A6, Atlanta, GA 30333 USA; Jhpiego, an affiliate of Johns Hopkins University, Washington, DC USA; Peace Corps, West Coast Region, San Francisco, CA USA; Zambia National Malaria Control Centre, Lusaka, Zambia; Bayer (Pty) Ltd, Isando, South Africa; Success Academy Charter Schools, Brooklyn, NY USA; Society for Family Health, Lusaka, Zambia; Center for Global Health, Division of Parasitic Diseases and Malaria, Centers for Disease Control and Prevention, Atlanta, GA USA; National Center for Enteric, Zoonotic, and Infectious Diseases, Centers for Disease Control and Prevention, Atlanta, GA USA; Center for Global Health, Entomology Branch, Centers for Disease Control and Prevention, Atlanta, GA USA; Center for Global Health, Polio Eradication Branch, Centers for Disease Control and Prevention, Atlanta, GA USA

**Keywords:** Long-lasting insecticidal nets, Durability, Attrition

## Abstract

**Background:**

A key goal of malaria control is to achieve universal access to, and use of, long-lasting insecticidal nets (LLINs) among people at risk for malaria. Quantifying the number of LLINs needed to achieve and maintain universal coverage requires knowing when nets need replacement. Longitudinal studies have observed physical deterioration in LLINs well before the assumed net lifespan of 3 years. The objective of this study was to describe attrition, physical integrity and insecticide persistence of LLINs over time to assist with better quantification of nets needing replacement.

**Methods:**

999 LLINs distributed in 2011 in two highly endemic provinces in Zambia were randomly selected, and were enrolled at 12 months old. LLINs were followed every 6 months up to 30 months of age. Holes were counted and measured (finger, fist, and head method) and a proportional hole index (pHI) was calculated. Households were surveyed about net care and repair and if applicable, reasons for attrition. Functional survival was defined as nets with a pHI <643 and present for follow-up. At 12 and 24 months of age, 74 LLINs were randomly selected for examination of insecticidal activity and content using bioassay and chemical analysis methods previously described by the World Health Organization (WHO).

**Results:**

A total of 999 LLINs were enrolled; 505 deltamethrin-treated polyester nets and 494 permethrin-treated polyethylene nets. With 74 used to examine insecticide activity, 925 were available for full follow-up. At 30 months, 325 (33 %) LLINs remained. Net attrition was primarily due to disposal (29 %). Presence of repairs and use over a reed mat were significantly associated with larger pHIs. By 30 months, only 56 % of remaining nets met criteria for functional survival. A shorter functional survival was associated with having been washed. At 24 months, nets had reduced insecticidal activity (57 % met WHO minimal criteria) and content (5 % met WHO target insecticide content).

**Conclusions:**

The median functional survival time for LLINs observed the study was 2.5–3 years and insecticide activity and content were markedly decreased by 2 years. A better measure of net survival incorporating insecticidal field effectiveness, net physical integrity, and attrition is needed.

## Background

A key goal of malaria control is to achieve universal access to, and use of, long-lasting insecticidal nets (LLINs) among people at risk for malaria [1]. In striving for universal coverage, there has been rapid scale-up of LLIN programmes in sub-Saharan Africa, as demonstrated by the increase in numbers of LLINs delivered by manufacturers to that region; from only six million LLINs in 2004 to 136 million LLINs in [2]. In 2014, an estimated 200 million LLINs were financed by donors [2].

Quantifying the number of LLINs needed to achieve and maintain universal coverage requires knowing when worn or lost nets need to be replaced. The lifespan of nets is thought to be 3 years with adequate insecticidal activity [3], but longitudinal studies have observed physical deterioration in LLINs well before 3 years [4, 5] and maintenance of insecticidal activity may be affected by varying conditions and handling practices (e.g. washing) [6]. Usage patterns have the potential to impact survival; worn nets might be thrown out or repaired and kept. A World Health Organization (WHO) technical expert group has emphasized the need for local data on LLIN durability in the field to make programmatic decisions [7].

In Zambia, where malaria transmission occurs year round and about 90 % of the population are at risk for malaria, 68.1 % of households owned at least one LLIN and 48.9 % of all surveyed slept under the LLIN the night before in [8]. As a step closer to universal coverage, Zambia planned mass campaigns in 2014, with replacement of worn nets afterwards through a combination of routine distribution and more mass campaigns. To quantify the numbers of nets needing replacement, a cross-sectional LLIN durability study in Zambia examined LLINs aged 2–4 years to look at LLIN durability [9]. The study found that the youngest (2–2.5 years) and oldest (greater than 3.5–4 years old) nets had equivalent large median total hole surface areas, but LLINs aged 3–3.5 years had a significantly lower median total hole surface area than the youngest. These findings suggested that physical deterioration of nets occurred prior to 2 years, and that perhaps very worn nets were thrown out, resulting in either stable or lower median total hole areas in the older nets. This survivorship bias results in a lowering or improvement in the median total hole area over time, and has also been observed in other studies [10, 11]. A recommendation from this study was to do a prospective net durability study starting prior to 2 years of net age, to better describe physical deterioration and attrition of nets; information that will be used to quantify net replacement needs. Furthermore, there is limited information on how local handling practices in Zambia might affect LLIN insecticidal activity.

The objective of this longitudinal study was to describe the durability of LLINs under local operational conditions in two provinces in Zambia in terms of attrition, physical integrity, and insecticidal activity. PermaNet 2.0 deltamethrin-treated polyester LLINs (Vestergaard-Frandsen) and Olyset permethrin-treated polyethylene (Sumitomo Chemical Co.) nets are the most widely distributed nets in Zambia to date, so a secondary objective of this study was to compare durability of PermaNet to Olyset nets.

## Methods

### Setting

This study was conducted in Luapula and Northern Provinces, two provinces selected because they have some of the highest malaria parasite prevalences among children under 5 years of age, at 32.1 and 23.7 %, respectively [6]. Districts within these provinces that received LLINs during a 2011 mass distribution campaign were selected based on presence of United States Peace Corps Volunteers who assisted in the implementation of the study, and included Mansa, Mwense, Nchelenge, and Samfya Districts in Luapula Province; and Kasama, Mbala, Luwingu, and Mpika Districts in Northern Province. LLINs had been distributed in a door-to-door campaign with Northern Province receiving only Olyset nets and Luapula Province receiving PermaNet nets. The districts were a mix of rural and peri-urban areas.

### Selection of nets

Villages within these districts were chosen if they participated in the 2011 mass distribution campaign, and if the village was within the catchment area of Peace Corps Volunteers who did the data collection. Every other household was chosen from a complete roster of households within these villages. If more than one LLIN from the mass distribution in 2011 was present, one LLIN was randomly selected for follow up in the study. Because there is no validated definition of LLIN failure, sample size was calculated using a “worst case” 50 % net failure from one time point to another. To detect a 10 % difference between follow-up time points, assuming a 5 % two-sided alpha, with 80 % power, a total of 1000 LLINs were needed. As only one brand of LLIN was distributed in each province, we aimed for 500 Olyset in Northern Province and 500 Permanet LLINs in Luapula Province.

### Questionnaire and examination of net

The study began 1 year after the 2011 mass distribution, so LLINs selected were already 12 months of age. At enrolment, these LLINs were marked with an identification number with permanent black marker, then followed prospectively every 6 months at 18, 24, and 30 months of age. At the initial and follow-up visits, interviews were conducted with a household adult aged 18 years or older using a standard questionnaire asking about the use, care, and, if the LLIN was not present, reason for attrition. Also at each visit, nets were examined for repairs, burns, holes, and tears. Holes were counted and measured in the field using the thumb, fist, head method described by the WHO [3]. Hole sizes were approximated as follows: smaller than a thumb (0.5–2 cm diameter), larger than a thumb but smaller than a fist (2–10 cm diameter), larger than a fist but smaller than a head (10–25 cm diameter), and larger than a head (>25 cm diameter). Then, proportional hole index (pHI) was calculated by weighting each hole by its size and summing as described elsewhere [12]. The weights were derived by taking approximations of hole area for each size category (1.23 cm^2^ for holes categorized as “less than a thumb”, 28.28 cm^2^ for holes “larger than a thumb but smaller than a fist”, 240.56 cm^2^ for holes “larger than a fist but smaller than a head”, and 706.95 cm^2^ for holes larger than a head), and dividing these hole areas by the smallest hole area (1.23 cm^2^). For the smallest through largest categories of holes, the weights were 1, 23, 196, and 576, respectively. The location of holes was also documented (roof, upper half, lower half).

### Net collection and preparation

At the initial visit when nets were 12 months of age and at the follow up visit at 24 months of age, random samples of nets were collected for bioassays and chemical analysis. A total of 74 LLINs were collected; 36 at the initial visit (18 in each province), and 38 at the 24-month visit (18 in one province, 20 in the other). Sections were cut from five areas of the net including the roof and side (top ¼, upper-middle ¼, lower-middle ¼, lower ¼); five 30 × 30 cm sections for bioassays, and five 10 × 10 cm samples for chemical analysis. Samples were individually wrapped in foil, and placed in a black plastic bag for transport and storage.

### Bioassay methods

Cone bioassays following the WHO methodology as described elsewhere [12] and using insectary-reared Kisumu strain, susceptible *Anopheles gambiae* mosquitoes were conducted at the insectary of the National Malaria Control Centre in Lusaka, Zambia. However, the methods were ultimately modified to use fewer mosquitoes due to limited availability of mosquitoes at the insectary. Each section of net was tested twice with two cones, five mosquitoes under each cone, instead of the WHO-recommended four cones, for a total of 20 mosquitoes per section of net. An untreated net was used as a control. The proportion of mosquitoes knocked down at 60-minutes (KD60) post exposure and the proportion of mosquitoes dead at 24 h (mortality) were calculated.

### Chemical analysis

For the initial samples of nets at 12 months, only four specimens (from the roof, top 1/4, upper-middle 1/4, lower-middle 1/4) were analysed, with the sample from the bottommost location on the net being unavailable. For the nets at 24 months, specimens from all five locations of the net were analysed. The specimen set from each net was analysed as a group to yield an average value of insecticide concentration for the net. Chemical analysis was based on methods published by the Collaborative International Pesticides Analytical Council (CIPAC) [13, 14].

Deltamethrin analysis of PermaNet 2.0 net samples was based on CIPAC method 333 [14]. Each specimen set was weighed and added to a 125 ml Erlenmeyer flask fitted with a polytetrafluoroethylene-lined screw cap. Precisely 50 ml of an extraction solvent, consisting of 80/20 (v/v) isooctane/1,4-dioxane, was added to the flask. The flask was then sonicated for 15 min followed by 30 min of agitation in a shaker bath at 25 °C and 155 cycles/min. Approximately 1.5 ml of the extract was then transferred to a chromatographic sample vial using a glass syringe fitted with a 0.45 μm reconstituted cellulose syringe filter. High performance liquid chromatography (HPLC) was conducted on the extract using an Agilent 1200 HPLC equipped with a 150 × 4.6 mm (i.d.) Ascentis Si 5 μm column held at 40 °C. The mobile phase was 94/6 (v/v) isooctane/1,4-dioxane pumped at 1.5 ml/min. A UV detector set to 254 nm was used to detect analyte peaks. For each extract, three injections of 20 μl were made, averaged, and the results compared to those of two samples containing known deltamethrin concentrations (i.e. external standards).

Permethrin analysis of Olyset net samples was based on CIPAC method 331 [13]. Each specimen set was weighed and placed in a 100 ml round-bottom boiling flask, followed by heptane (50 ml) and triphenyl phosphate internal standard (5.0 ml of known concentration in heptane). The flask was fitted with a reflux condenser and heated to boiling for 45 min. After cooling, approximately 1.5 ml of the extract was transferred to a chromatographic sample vial using a glass syringe fitted with a 0.45 μm reconstituted cellulose syringe filter. Gas chromatography (GC) was conducted using an Agilent 6890 N chromatograph fitted with a 30m × 0.25 mm (i.d.) fused silica DB-1 capillary column coated with 0.25 μm cross linked polydimethylsiloxane stationary phase. Ultra high purity nitrogen (1.2 ml/min) was used as the carrier gas. Extract (1 μl) was injected into the column inlet using a split flow rate of 96.1 ml/min. Injector port, column oven, and detector temperatures were 265, 240, and 300 °C, respectively. Flame ionization was used for analyte detection. Two injections were used for each sample and the results averaged. Permethrin concentration was calculated by comparing permethrin/triphenyl phosphate peak area ratios against a calibration curve generated from solutions containing known permethrin/triphenyl phosphate mass ratios.

### Statistical analyses

All analyses were done using SAS^®^ v9.3 (SAS Institute, Cary, North Carolina, USA). Baseline characteristics such as net use, presence of holes and repairs, and reasons for LLIN loss were summarized with simple frequencies. Medians and interquartile range (IQR) were calculated for continuous variables that were not normally distributed. Then, functional LLIN survival was examined looking first at attrition. LLINs gone due to destruction, discarding, or use for other purposes were classified as LLINs with “known” reasons for attrition, and these LLINs were used in the numerator to estimate LLIN attrition. LLINs lost for other reasons such as being given away, used in a different location, stolen, no longer being used because a new LLIN was received, or lost to follow up (ex: family moved or not home), were categorized as “outcome unknown” and were not included in the denominator when calculating attrition. The formula for functional survival was:functional survival = (nets in “good” or “damaged” condition as defined by pHI)/(All nets present + nets missing due to known reasons).

Furthermore, to check representativeness of LLINs with known outcomes, baseline characteristics of nets with known outcomes were compared to nets with unknown outcomes using Chi square tests.

The other component of LLIN survival, physical integrity, was examined. The geometric mean (GM) and 95 % confidence intervals (CI) of the pHI were calculated and compared using t-tests for significance for nets at different ages and then stratified by LLIN type. This calculation was done for all LLINs in the study, and then for only the subset of LLINs present at all follow-up visits to better examine changes in median pHI over time in the same net. Then, using pHI values, nets were categorized as good (0–64), damaged (65–642), or too torn (>643) as suggested by WHO [7] and these proportions were compared by LLIN age and type.

The relationship between pHI and factors that might influence hole formation was then examined. Generalized estimating equation models of log-transformed pHI were done, and independent variables of interest were factors such as LLIN age, net type, burns, washing, and repair.

To examine if a particular part of the net was more prone to hole development, the geometric mean 95 % CIs of pHI values were calculated for different areas of the net—the roof, upper half, and lower half. Generalized estimating equations were also done with log-transformed pHIs to examine hole development by area of the net. Then, proportion of LLINs functionally surviving was calculated for each follow-up time point. Nets present at a particular time point for follow up and nets with a known reason for attrition were included in the denominator. The numerator was the number of LLINs that were present at that time point and classified as “good” or “damaged.” LLINs in the “outcome unknown” category were censored at time of net loss. These proportions and their confidence intervals were plotted by years since distribution, and compared against reference LLIN survival curves provided by the WHO [7]. The failure endpoint was therefore defined as either an LLIN classified as “too torn” or an LLIN having a known reason for attrition. Then, a Kaplan–Meier survival analysis was done and used to estimate overall median survival time, and to compare median survival time between net brands. Using the same definitions for censoring and failure endpoint, survival analysis using Cox proportional hazards (PROC PHREG in SAS v9.3) was also done to examine factors that might affect survival such as LLIN type, use the night before the survey, having ever been repaired, having ever been washed, and use over a reed mat. For these survival analyses, if a net was missing an observation point between the initial and a subsequent follow-up visit (for example, an LLIN present at enrolment, missing at 18 months of age, present at 24 months of age), the outcome status of the net was assessed at its last available time point.

For the bioassay results, geometric means and 95 % CIs were calculated for KD60 and mortality at 24 h, and compared with significance testing via t-tests. While there is no cutoff for net failure for bioassay results, the WHO Pesticide Evaluation Scheme (WHOPES) criteria for optimal bioefficacy are ≥95 % KD60 or ≥80 % mortality in nets that have had at least 20 washes and 3 years of use [12]. Proportion of nets with meeting these optimal bioefficacy levels of KD60 and mortality were calculated. A minimal bioefficacy criteria of ≥75 % KD60 or ≥50 % mortality has been used in the field [15], so proportion of nets meeting this minimal criteria was also calculated. Using Chi squared tests, proportions of nets meeting optimal and minimal bioefficacy results were compared at 12 and 24 months, and further stratified by net type. The bioassay results were stratified by LLIN type, age, and history of washing. The chemical analysis results were summarized as median insecticide content expressed in both mg/m^2^ and g/kg. Target insecticide content as recommended by WHO was 55 mg/m^2^ (or 1.8 g/kg for 75 denier net, and 1.4 g/kg for 100 denier net) deltamethrin used in PermaNet 2.0 nets, and at least 20 g/kg of permethrin used in Olyset nets [16, 17]. Results for chemical analysis were compared for nets of different ages and history of washing. Then, to describe the relationship between bioefficacy and chemical content in the study nets, the correlation between bioassay and chemical analysis results was examined by using R (R package version 1.4.8, Stanford, California, USA) to create McFadden probit models to obtain a pseudo R-squared value.

The protocol for this study was approved by investigational review boards at the US Centers for Disease Control and Prevention and the Tropical Disease Research Centre in Zambia. Written consent was obtained, using a consent form that had been translated to the local language, from an adult over the age of 18 years old at participating households.

## Results

### Characteristics at enrollment and study completion

A total of 999 LLINs were included in the study; 499 PermaNet and 500 Olyset nets. Baseline characteristics of LLINs upon enrollment at 12 months of age by LLIN brand are summarized in Table 1. A slightly larger proportion of PermaNet nets (93.6 %) were used the night before the survey versus Olyset (90.2 %) nets, and this difference was statistically significant. The physical integrity of the LLINs were similar between LLIN types with holes present in 67.9 % of PermaNet and 70.6 % of Olyset nets, and burns present in 6.6 % of PermaNet and 4.4 % of Olyset nets. The care of nets differed significantly in terms of washing, but did not differ in terms of repairs. A higher proportion of PermaNet (85.8 %) nets had ever been washed compared to Olyset (79.0 %) nets. Presence of repairs were equally rare in both types of nets, found in 7.2 % of PermaNet and 6.4 % of Olyset nets, and among nets with repairs present, the overall median number of repairs was 1 (interquartile range 1–2) and was similar between net types. The use of the net over a reed mat, found in a previous study to be associated with holes [9], was more common in Olyset (30.5 %) than PermaNet nets (18.9 %).

**Table 1:** Baseline characteristics of LLINs enrolled by net type

Characteristic	Permanet N = 499 n (%)	Olyset N = 500 n (%)	All LLINs N = 999 n (%)
ITN used last night*	466/498 (93.6)	449/498 (90.2)	915/996 (91.9)
Presence of any holes	339 (67.9)	353 (70.6)	692 (69.3)
Presence of burns	33 (6.6)	22 (4.4)	55 (5.5)
Median number of burns (interquartile range [IQR])	3 (1–5)	2 (1–3)	2 (1–5)
Presence of repairs	36 (7.2)	32 (6.4)	68 (6.8)
Median number of repairs (IQR)	1 (1–2)	1 (1–2)	1 (1–2)
Ever washed*	362/422 (85.8)	347/439 (79.0)	709/861 (82.3)
Used over a reed mat*	93/493 (18.9)	147/482 (30.5)	240/975 (24.6)

* Permanet versus Olyset, Chi square p ≤ 0.05

Of the 999 LLINs, the numbers of LLINs available for follow up at 18, 24, and 30 months of age were 721, 540, and 325, respectively. Ultimately, only 325 LLINs completed the follow-up period, 74 were taken for chemical analysis, and 600 did not complete follow up; reasons for loss are detailed in Table 2, and did not differ by LLIN type. Of the LLINs not completing follow up, a total of 381 (63.5 % of nets gone) had an unknown durability outcome due to being given away, stolen, sold, lost, used in a different location, family moved or not home during follow up, or with no information on why the net was not available for follow up (n = 80, 13.3 % of nets gone). Including those that completed the study, outcomes were known for a total of 544 LLINs. When comparing baseline characteristics of LLINs with unknown and known outcomes, there were no significant differences including: LLIN type, presence of any holes, ever having been repaired, ever having been burned, having been used the night before, ever been washed, use the night before, and use over a reed matt.

**Table 2:** Reasons for LLIN loss between enrollment and end of follow up period (N = 600)

Reason	n (%)
Damaged and thrown away	174 (29.0)
Used in a different location	66 (11.0)
Given away	38 (6.3)
Used for a different purpose	36 (6.0)
Threw away LLIN because received new LLIN^a^	25 (4.2)
Stolen	11 (1.8)
Destroyed in a fire	9 (1.5)
Sold	3 (0.5)
Other^b^	158 (26.3)
Unknown reasons	80 (13.3)

^a^“Received new LLIN” means that the owner of the net received a new LLIN through a routine distribution method (such as via antenatal care clinics, or childhood vaccination clinics), and threw away the LLIN being followed in the study

^b^Other includes: family moved (61), family not home (77), LLIN lost by owner (16), and refusal to continue to participate (4)

### Attrition

Of the 544 nets with known outcome, the overall attrition from the beginning of the study to the end was 40.3 % (n = 219). For these nets with a known outcome, the median age at which nets were no longer available for follow up was 18 months (interquartile range 12–24 months). Reasons that accounted for this attrition included being destroyed due to fire (n = 9, 4.1 %), being disposed of reportedly due to damage (n = 174, 79.5 %), and used for a different purpose (n = 36, 16.4 %). When comparing baseline characteristics of nets that survived versus those that did not in univariate analysis, surviving nets were more likely to be PermaNet brand (unadjusted odds ratio [uOR] 1.81, 95 % confidence interval [CI] 1.28–2.57), less likely to have had any holes at baseline (uOR 0.40, 95 % CI 0.27–0.60), more likely to have been used the night before (uOR 2.27, 95 % CI 1.17–4.41), and less likely to have been used over a reed mat (uOR 0.51, 95 % CI 0.34–0.77). There were no significant differences among nets that survived versus those that did not in terms of ever having been repaired, having been burned, or ever having been washed at baseline.

### Physical integrity

Of the 325 nets present for the whole study period (i.e. present at the first and last follow up visits), only 274 were identified at every follow-up visit, and could be used to look at changes in holes across all time points. For these nets with complete data, 137 were PermaNet nets and 137 were Olyset nets; the proportion of nets with any holes, the GMs of the pHI and associated 95 % CIs are stratified by age and net type in Table 3. The GMs of pHI increased with age, as seen in the non-overlapping 95 % CIs. These findings are contrasted with the GMs of pHI that would be found if all LLINs, including those that eventually dropped out, were included in the calculations (Table 3). When all LLINs were included in the calculation, LLINs at 24 and 30 months were found to not have significantly different pHIs.

**Table 3:** Comparison of geometric means (GM) of proportionate hole index (pHI) of LLINs at different ages

All LLINs in the study	12 months	18 months	24 months	30 months
	n = 999	n = 721	n = 520	n = 325
Any holes n (%)	692 (69.3)	551 (76.4)	448 (86.2)	290 (89.2)
GM pHI (95 % confidence interval [CI])	15.5 (13.1–18.4)	33.8 (27.3–41.6)	77.8 (61.9–97.9)	102.8 (77.5–136.3)
GM pHI Permanets^®^ (95 % CI) n = 499	12.6 (9.8–16.0)	30.0 (22.3–40.2)	91.3 (67.4–123.5)	93.6 (64.0–136.6)
GM pHI Olysets^®^ (95 % CI) n = 500	19.1 (15.0–24.4)	37.7 (27.9–50.8)	66.5 (47.1–93.7)	115.0 (75.3–175.5)
Only LLINs present at all follow-up visits, n = 274				
Any holes n (%)	165 (60.2)	196 (71.5)	239 (87.2)	271 (91.6)
GM pHI (95 % CI)	6.7 (4.8–9.3)	20.5 (14.6–28.5)	65.3 (48.2–88.5)	122.7 (91.1–165.3)
GM pHI Permanets^®^ (95 % CI) n = 137	6.3 (3.9–9.9)	22.1 (13.9–35.0)	73.6 (49.1–109.9)	124.4 (82.2–188.1)
GM pHI Olysets^®^ (95 % CI) n = 137	7.2 (4.5–11.2)	19.0 (11.7–30.5)	58.0 (36.7–91.3)	121.0 (78.7–185.7)

The proportion of LLINs in good, damaged, or too torn conditions at different ages of follow up are summarized in Table 4. The proportion of LLINs in the “too torn” category increased with age. When taking into account all nets, the proportion of LLINs that were “too torn” increased 9.6–29.5 % from 12 to 30 months of age. When only looking at LLINs present at all follow-up visits, 3.7 % of nets versus 31.4 % of nets were “too torn” at 12 and 30 months, respectively. There were no significant differences in pHI categories when comparing LLIN types.

**Table 4:** Proportion of LLINs in good, damaged, or too torn conditions at different ages, as defined by proportionate hole index (pHI)

Category as defined by pHI	12 months n (%)^a^	18 months n (%)	24 months n (%)	30 months n (%)
All nets	n = 999	n = 721	n = 520	n = 325
Good (0–64)	656 (65.7)	381 (52.8)	200 (38.5)	117 (36.0)
Damaged (65–642)	247 (24.7)	205 (28.4)	188 (36.2)	112 (34.5)
Too torn (>643)	96 (9.6)	135 (18.7)	132 (25.4)	96 (29.5)
LLINs present at all follow-up visits, n = 274^a^				
Good (0–64)	209 (76.3)	163 (59.5)	113 (41.2)	89 (32.5)
Damaged (65–642)	55 (20.1)	79 (28.8)	106 (38.7)	99 (36.1)
Too torn (>643)	10 (3.7)	32 (11.7)	55 (20.1)	86 (31.4)

^a^No significant differences between Permanets^®^ and Olysets^®^

Repeat measures ANOVA using the outcome of pHI as a continuous variable showed a statistically significant (p < 0.0001) effect of age on increasing pHI. For every 6 months of increasing age, there was an approximately 1 unit log increase in pHI. There were no significant differences in GMs of pHI when comparing PermaNet nets to Olyset nets of the same age. Factors over the lifetime of the net that were significantly correlated with pHI on crude analysis included having ever been repaired and being used over a reed mat (Table 5). Because PermaNet nets were in one province while Olyset nets were in another, and since there may be unaccounted for differences in how LLINs are treated by province due to local customs, or differences in settings between provinces, the final model controlled for LLIN type. When controlling for LLIN type, the relationship between pHI and age of LLIN are modified significantly by an LLIN ever having been repaired or being used over a reed mat (p < 0.0001 and p = 0.0130, respectively) with both factors associated with larger pHIs.

**Table 5:** Lifetime factors of an LLIN affecting relationship of age of LLIN and proportionate hole index (pHI)

Factor	Unadjusted repeat measures ANOVA (p value)	Adjusted repeat measures ANOVA (p value)
LLIN type	0.7599	0.6649
Net used last night	0.1835	
Ever repaired	<0.0001	<0.0001
Any burns	0.0867	
Ever washed	0.3523	
Used over a reed mat	0.0036	0.0130

**Table 6:** Comparison of geometric means (GM) of proportionate hole index (pHI) of different locations of LLINs by age and LLIN type for LLINs with complete follow-up (n = 274)

Age in months	Part of LLIN	GM (95 % confidence intervals [CI])		
		PermaNet^®^	Olyset^®^	All nets
12	Roof	0.3 (0.1–0.6)	0.3 (0.1–0.5)	0.3 (0.1–0.5)
	Upper half	0.4 (0.2–0.8)	0.5 (0.2–0.9)	0.5 (0.3–0.7)
	Lower half	0.8 (0.5–1.3)	1.0 (0.6–1.5)	0.9 (0.6–1.3)*
	Seams	0.2 (0.02–0.3)	0.1 (0.007–0.2)	0.1 (0.05–0.2)
18	Roof	0.8 (0.4–1.2)	2.6 (1.3–4.6)	1.3 (0.8–1.9)
	Upper half	1.1 (0.6–1.7)	2.1 (1.2–3.2)	1.5 (1.1–2.1)
	Lower half	14.3 (8.9–22.6)	13.3 (8.4–20.8)	13.8 (9.9–19.0)*
	Seams	0.5 (0.2–1.0)	1.1 (0.5–1.7)	0.8 (0.5–1.2)
24	Roof	1.2 (0.7–1.9)	3.1 (1.9–4.9)	2.0 (1.4–2.8)
	Upper half	3.3 (2.1–5.1)	5.0 (3.1–7.8)	4.1 (2.9–5.6)
	Lower half	34.4 (22.2–53.1)	26.6 (16.8–41.9)	30.3 (22.0–41.4)*
	Seams	1.1 (0.6–1.8)	1.0 (0.5–1.7)	1.1 (0.6–1.5)
30	Roof	0.1 (1.4–3.3)	4.6 (2.9–7.1)	3.2 (2.3–4.4)
	Upper half	7.6 (4.7–12.0)	7.9 (4.9–12.5)	7.9 (5.6–10.7)
	Lower half	54.0 (34.2–85.1)	57.7 (37.1–89.4)	55.8 (40.6–76.6)*
	Seams	1.0 (0.5–1.6)	1.5 (0.8–2.4)	1.2 (0.8–1.7)

* lower half versus other parts of the LLIN, p < 0.001

When examining holes by location on the net for the LLINs with complete follow up (Table 6), the lower half of the net had significantly higher GMs for pHI than other parts of the net at any age (p < 0.001). By the end of the study, nets at 30 months of age had a GM pHI of 55.8 (95 % CI 40.6–76.6) at the lower half of the net, 7.9 (95 % CI 5.6–10.7) at the upper half, 3.2 (2.3–4.4) at the roof, and 1.2 (0.8–1.7) at the seams. The GMs of pHIs stratified by location on the net were similar between PermaNet and Olyset nets. Furthermore, nets used over a reed mat had significantly larger holes at the bottom (p = 0.012).

### Survival curves and survival analysis

**Fig. 1:**
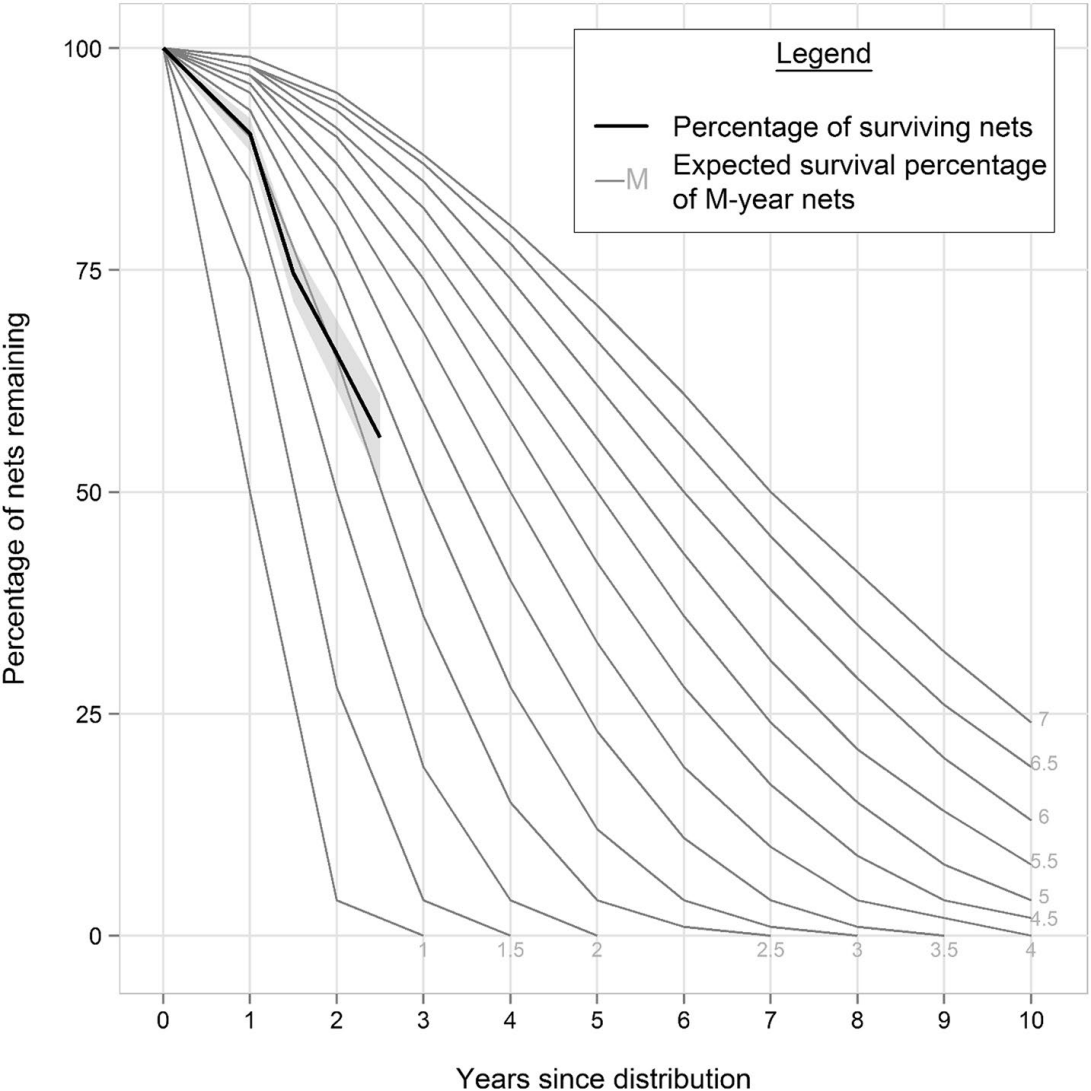
Proportion of LLINs functionally surviving

The proportion of LLINs functionally surviving at different time points relative to reference survival curves are shown in Fig. 1. At the initial visit at 12 months after net distribution, 90.4 % of LLINs survived. By 30 months, only 56.1 % of nets had survived. By extrapolation, the median survival time (time after distribution at which 50 % of nets are still around and serviceable) is estimated to be between 2.5–3 years in the study nets.

The adjusted proportional hazards model indicated that longer survival times were associated with having been used the night before (hazard ratio [HR] 0.31, p < 0.001), and having ever been washed (HR 0.61, p < 0.01). Nets that had ever been repaired had a shorter survival time (HR 1.38, p < 0.002). However, nets hung over a reed mat had similar survival times to those hung over other types of sleeping spaces. There were no differences in survival between LLIN types.

### Bioassay results

A total of 74 LLINs were collected for bioassay testing (Table 7). At 12 months, the KD60 had a GM of 85.7 % (95 % CI 80.9–90.9) and at 24 months, the KD60 had fallen significantly to 35.7 % (95 % CI 22.3–56.7) (p < 0.001). The KD60 was significantly higher in PermaNet versus Olyset nets at 12 months at 92.6 % (95 % CI 87.6–97.8) and 79.4 % (95 % CI 71.6–88.0), respectively (p = 0.01). By 24 months, KD60 decreased with no significant differences between LLIN types (p = 0.45). The GM of mortality at 24 h was 64.8 % (95 % CI 54.7–76.8) at 12 months and significantly lower at 41.0 % (95 % CI 27.9–60.1) at 24 months (p = 0. 04). At 12 months of age, Olyset nets killed significantly (p < 0.001) fewer mosquitoes (47.1 %, 95 % CI 33.6–65.7) than PermaNet nets (89.1 %, 95 % CI 83.2–95.4), but by 24 months, there was no difference (p = 0.22) in functional mortality. Untreated nets used as controls had no effect on the mosquitoes with zero mortality and knockdowns.

**Table 7:** Cone bioassay results using *Anopheles gambiae s.s* (Kisumu strain) by net type and age

Net age at follow-up	PermaNet^®^	Olyset^®^	All nets
Geometric mean of % of mosquitoes knocked down at 60 min of exposure (n) GM 95 % CI			
12 months	(18) 92.6 87.6–97.8	(18) 79.4 71.6–88.0	(36) 85.7 80.9–90.9
24 months	(18) 29.6 12.3–69.1	(20) 42.2 27.2–65.3	(38) 35.7 22.3–56.7
Average % mortality at 24 h of exposure			
12 months	(18) 89.1 83.2–95.4	(18) 47.1 33.6–65.7	(36) 64.8 54.7–76.8
24 months	(18) 31.9 14.9–67.0	(20) 51.4 38.7–68.2	(38) 41.0 27.9–60.1

*GM* geometric mean, *CI* confidence interval

The proportion of nets meeting optimal and minimal effectiveness criteria are summarized in Table 8 and stratified by net type. At 12 months, the proportion of nets meeting optimal effectiveness criteria was only 55.6 % (95 % CI 39.3–71.8), and the proportion fell to 34.2 % (95 % CI 19.1–51.4), by 24 months but this change was not statistically significant (p = 0.06). Comparing LLIN types, optimal effectiveness criteria was met by a significantly higher percentage of PermaNets 77.8 % (95 % CI 58.6–96.7) versus Olyset nets 33.3 % (95 % CI 11.6–55.1) at 12 months (p < 0.01), but there was no difference in proportions meeting optimal effectiveness criteria at 24 months. Minimal effectiveness criteria was met by 88.9 % (95 % CI 78.6–96.9) of LLINs, and this percentage decreased significantly by 24 months (65.8 %, 95 % CI 50.7–80.9) (p = 0.02). When comparing types of LLINs, the proportion of PermaNet LLINs meeting minimal effectiveness standards at 12 months was significantly greater (100 %, 95 % CI 100.0–100.0) than Olyset nets (77.8 %, 95 % CI 58.6–97.0) (p = 0.03), but at 24 months, there was no difference between net types.

**Table 8:** Proportion of nets meeting optimal (KD60 ≥95 % or mortality ≥80 %) and minimal WHO criteria (KD60 ≥75 % or mortality ≥50 %)

Optimal effectiveness (n) %, 95 % CI			
	PermaNet^®^ (n) %, 95 % CI	Olyset^®^ (n) %, 95 % CI	All nets (n) %, 95 % CI
LLIN age			
12 months	(14) 77.8, 58.6–96.7^†^	(6) 33.3, 11.6–55.1	(20) 55.6, 39.3–71.8
24 months	(7) 38.9, 16.4–61.4	(6) 30.0, 9.9–50.1	(13) 34.2, 19.1–51.4
Minimal effectiveness			
12 months	(18) 100.0, 100.0–100.0^†^	(14) 77.8, 58.6–97.0	(32) 88.9, 78.6–96.9
24 months	(11) 61.1, 38.6–83.6	(14) 70.0, 49.9–88.1	(25) 65.8, 50.7–80.9*

* All nets, 12 versus 24 months, p < 0.01

^†^PermaNet versus Olyset, p < 0.01

### Chemical analysis results

**Fig. 2:**
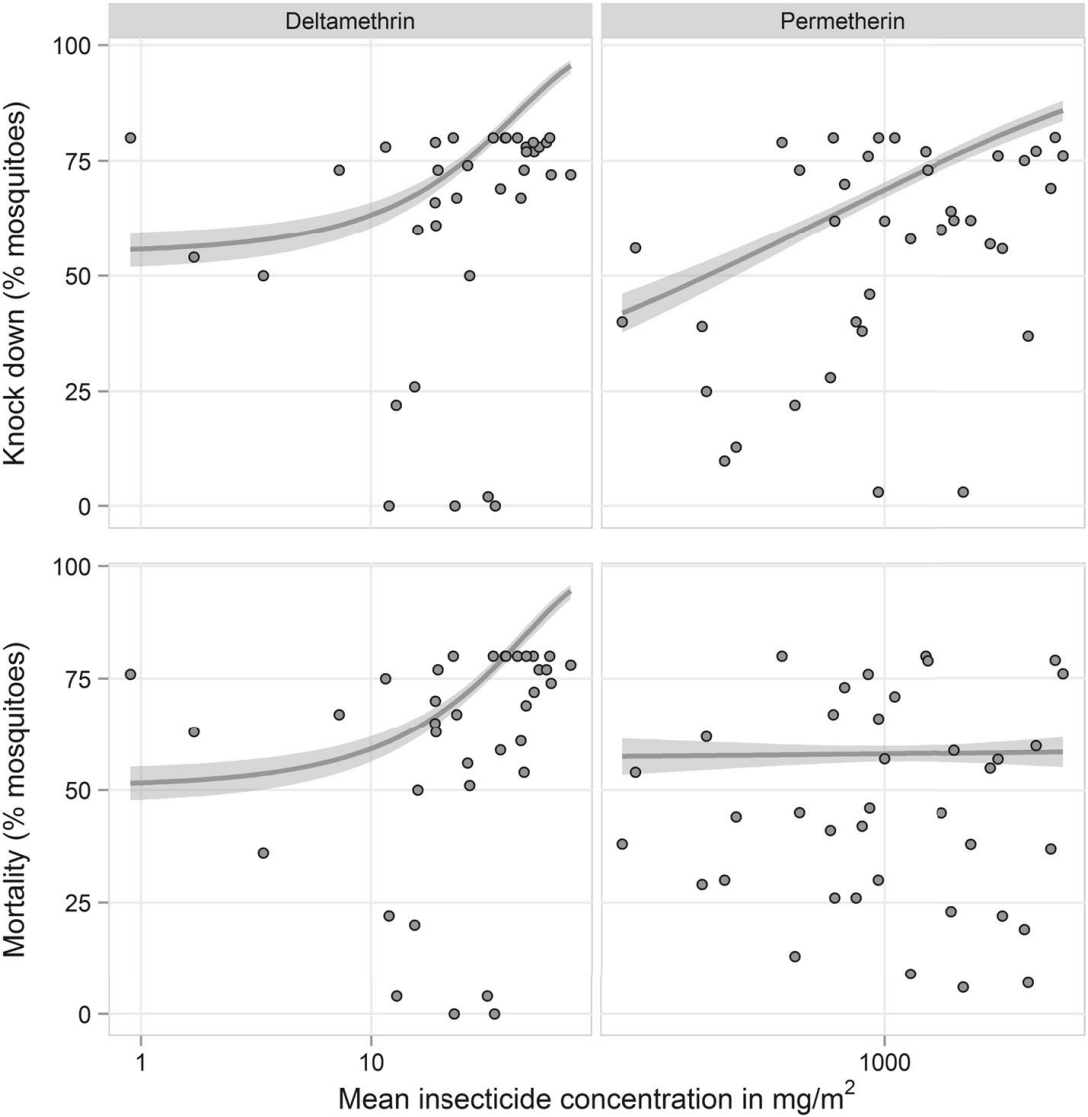
Correlation of chemical and bioassay results (95 % confidence intervals shown in *shaded area*)

At 12 months, 18 PermaNet and 18 Olyset nets underwent chemical analysis for insecticide content. At 24 months, 18 PermaNet and 20 Olyset nets were sampled. The median deltamethrin content of PermaNet nets at 12 months was 45.6 mg/m^2^ with IQR 34.0–53.7 mg/m^2^ (1.2 g/kg, IQR 1.0–1.6 g/kg) and at 24 months was less at 19.1 mg/m^2^ with IQR 12.0–26.3 (0.5 g/kg, IQR 0.3–0.7). The median permethrin content of Olyset nets at 12 months decreased at 24 months and was 1100.1 mg/m^2^ (IQR 1026.1–1152.5 mg/m^2^) (23.0 g/kg, IQR 21.5–24.3 g/kg) and 950.0 mg/m^2^ (IQR 859.4–989.8 mg/m^2^) (18.2 g/kg, IQR 16.1–19.1 g/kg), respectively. When comparing insecticide levels to target levels for new nets, at 12 months 83 % of Olyset nets were at or above the target threshold of 20 g/kg of permethrin and 22 % of PermaNet nets were at or above 55 mg/m^2^ of deltamethrin. By 24 months, these percentages were 5 % for Olyset nets and 0 % for PermaNet nets. When comparing nets that had ever been washed to those that had not been washed, there were no differences in insecticide content; this was observed for both PermaNet (p = 0.6710) and Olyset (p = 0.067) nets. The results of correlating bioassay data to chemical analysis data is shown in Fig. 2 for PermaNet and Olyset nets. For PermaNet nets, the pseudo R^2^ for the correlation between knockdown or mortality to deltamethrin content was 0.52 and 0.53, respectively. For Olyset nets, the correlation between permethrin content and knockdown or mortality had pseudo R^2^ = 0.62 and pseudo R^2^ = 0.59, respectively.

## Discussion

In Zambia, only a little more than half of LLINs distributed were functionally surviving by two and a half years, when taking into account both attrition and physical durability. There was no difference in functional longevity between PermaNet 2.0 and Olyset nets. Furthermore, by 24 months, almost all of the nets did not meet target concentrations of insecticides. Attrition was primarily caused by disposal; relatively few nets were found to be used for alternate purposes. As expected, the pHI as well as the proportion of nets in the “too torn” category increased over time. For every 6 months of increasing age, there was a one log unit difference in pHI; in other words, the deterioration with age is exponential, not linear, in terms of the pHI. Contrary to what other studies have found, the present study found no statistically significant difference in pHI between PermaNet 2.0 and Olyset nets [5, 18].

The observation of a propensity for hole development at the bottom part of the net has also been reported in other studies, and is thought to be due to how nets are secured in the sleeping space [4, 18, 19]. The findings of higher pHIs in nets used over a reed mat, and the association between use of a net over a reed mat and larger holes at the bottom of the net seems to support this explanation. It appears that tucking nets under reed mats causes damage to the lower half of the net. A previous cross-sectional study had not observed this association [9], but unlike a cross-sectional study, the prospective nature of this study allowed for observation of hole development prior to nets being thrown away. It is possible that nets with a reinforced lower half would improve longevity for LLINs, or that the recommendation to tuck nets under the mats should be reconsidered.

The complicated nature of net care behaviors and LLIN durability was observed in this study. Behaviors found to be associated with net longevity, such as washing the net, could be indicators of the net being valued by the owner, and therefore kept longer with less attrition. Use of the net the previous night could be indicator of net longevity. Net repair, a behaviour that might be expected to prolong physical integrity, was associated with larger pHI and shorter survival times. Mutuku et al., had similar findings, in which most nets with repairs were categorized as “ineffective nets” based on pHI [19]. Nets requiring repair are likely those in poor physical condition, and therefore have a shorter lifespan. There is limited evidence of the effect of repair on physical longevity, and the available studies on net repair are primarily qualitative [20–22]. The issue of repair and its effect on physical durability of a net needs further study before repair can be recommended as a way to prolong net life.

One behavior in net use that was found to be detrimental to physical integrity especially at the bottom portion of the net was the use of the net over a reed mat. It is likely that when tucking the net under the reed mat, the reeds abrade the net material. Since the net can be hung to reach the floor with reed mats, it might not be necessary to tuck the net under the reeds, preventing the net from being abraded, so the current recommendation to tuck the net under the reed mat needs to be more closely examined. However, when the outcome of functional survival was examined, the effect of using reed mats on the overall functional survival of the nets was not significant; likely because this outcome also takes into account net attrition.

The insecticidal effectiveness of the LLINs in the study were described using WHO measures of optimal effectiveness and minimal effectiveness criteria discussed by WHO, but unpublished [7, 12]; both of which have been applied in the field [15]. For both optimal and minimal effectiveness criteria, insecticidal activity trended downwards by 2 years of age, and Olyset nets seemed to have a significantly lower insecticidal activity at 12 months than PermaNet nets. The finding that insecticidal content did not differ between nets that had ever been washed versus those that had never been washed was surprising, but may be due to the lack of data to take into consideration the number of times a net had been washed, as well as how nets were washed (i.e. with or without soap). The true threshold for determining when insecticidal protection is inadequate has not been well defined. It has been suggested that LLINs with WHOPES approval, as both types of nets in the study have received, have evidence to support an assumption of 3 years of insecticidal protection [7]. Attempts to correlate insecticide levels with bioassay results seem to suggest levels much higher than 10 mg/m^2^ of deltamethrin are needed achieve between 75–95 % knockdown, and levels much higher than 1000 mg/m^2^ of permethrin are needed to achieve between 75–95 % knockdown. It was surprising to see that while permethrin levels increased, there was not a concomitant increase in percent mortality. One possibility is that associations between mortality and permethrin levels are seen at levels much higher than 1000 mg/m^2^ of permethrin. Kilian et al. correlated chemical and bioassay results for PermaNet nets using a much larger sample size and found that while knockdown rates and mortality dropped sharply when deltamethrin concentrations were below 15 mg/m^2^, concentrations of 4 mg/m^2^ were still associated with knockdown rates above 75 %, and a probability of at least 90 % that a net would have minimal effectiveness [6].

Of note, there is no definition of net failure that incorporates a combination of variables reflecting attrition, physical integrity, and insecticidal effectiveness of nets. Using current, but limited evidence, WHO has suggested calculating functional survival using both physical integrity and attrition [7], as was done in this study. However, it is still unknown how best to incorporate insecticidal effectiveness in a measure of net durability. The minimal effective concentration of insecticide in a net and how it would translate in terms of bioassay results is unknown, and difficult to determine with current methods that require removal and destruction of a small sub-sample of nets. A field test, allowing for prospective studies of insecticide effectiveness in a larger sample size of nets needs to be developed.

## Limitations

Monitoring of nets started 12 months after the nets were distributed. The WHO recommends that nets should be monitored starting at 6 months [3] after distribution to capture reasons for attrition and wear in the first year of use. The functional survival observed in this study does not account for the nets that don’t survive the first year. The prospective nature of this study may have resulted in participants keeping LLINs longer than they might have had they been unobserved. Therefore, attrition rates due to alternate uses of the net or disposal might be lower, and the physical condition observed in the nets might be worse than normally tolerated before net disposal. The net survival times observed in the study may be an overestimate of true net retention. There were also a large number of nets that were no longer available for follow up for unknown reasons, which has the potential to bias the results. However, when baseline characteristics of these nets were compared to those of nets that were available for the study or gone for known reasons, there were no differences. Of the nets that were followed, only PermaNet nets were in one province while only Olyset nets were in the other. Differences observed between net types such as use over a reed mat and washing of the net are likely reflective of differing provincial circumstances and customs. The findings regarding net repair were limited by a small sample size as LLINs in this study were infrequently repaired, consistent with observations of other studies [4, 18, 23].

The findings of insecticide content were possibly affected by delays between LLIN sampling and LLIN testing for both chemical analysis and bioassay results. Rearing of mosquitoes for the bioassay, as well as processing of samples for the chemical analysis resulted in delays as much as 1 year between collection and testing, and could have resulted in lower insecticide content findings. Samples were stored in a freezer to preserve the specimens as much as possible. Additionally, the attempts to compare chemical content and bioassay results were limited by a very small sample size. This might account for the lack of significance in the weak correlation observed between mortality of mosquitoes on bioassay and permethrin levels.

## Conclusions

Quantification of nets requiring replacement in Zambia should consider a lifespan consistent with the 2.5–3 year median survival time observed in the LLINs in this study, regardless of (Olyset or PermaNet) brand. However, the bioassay and the chemical analysis seemed to suggest that the insecticide activity and content decreased markedly by 2 years of age. More study is needed in the relationship between bioassay results, insecticide content, and how this translates to insecticide effectiveness in the field. Furthermore, a better measure of net survival incorporating insecticidal effectiveness, net physical integrity, and attrition is needed.

## Abbreviations

CI 95 %: confidence interval
CIPAC: collaborative International Pesticides Analytical Council
GM: geometric mean
KD60: proportion of mosquitoes knocked down at 60 min post-exposure
HPLC: high performance liquid chromatography
HR: hazard ratio
IQR: intraquartile range
LLIN: long-lasting insecticidal nets
pHI: proportional hole index
WHO: World Health Organization
WHOPES: World Health Organization Pesticide Evaluation Scheme
